# Tibial fracture surgery in elderly mice caused postoperative neurocognitive disorder via SOX2OT lncRNA in the hippocampus

**DOI:** 10.1186/s13041-023-01024-y

**Published:** 2023-04-25

**Authors:** Zhibin Xiao, Xiajing Zhang, Guangyao Li, Li Sun, Jiangjing Li, Ziwei Jing, Qingya Qiu, Guangxiang He, Changjun Gao, Xude Sun

**Affiliations:** 1 Department of Anesthesiology, The Second Affiliated Hospital of Air Force Medical University, Xi’an, 710032 Shaanxi China; 2 Department of Anesthesiology, The 986th Air Force Hospital, Xijing Hospital, The Fourth Military Medical University, Xi’an, 710032 Shaanxi China; 3 Institute of Medical Research, Northwestern Polytechnical University, Xi’an, 710032 Shaanxi China; 4 Department of Physiology and Pathophysiology, National Key Discipline of Cell Biology, The Fourth Military Medical University, Xi’an, 710032 Shaanxi China

**Keywords:** Postoperative neurocognitive disorder, Mitochondrial dynamics, Oxidative stress, SOX2OT, SOX2, Drp1

## Abstract

Increasing evidence indicates the major role of mitochondrial function in neurodegenerative disease. However, it is unclear whether mitochondrial dynamics directly affect postoperative neurocognitive disorder (PND). This study aimed to analyze the underlying mechanisms of mitochondrial dynamics in the pathogenesis of PND. Tibial fracture surgery was performed in elderly mice to generate a PND model in vivo. Cognitive behavior was evaluated 3 days post-surgery using novel object recognition and fear conditioning. A gradual increase in the SOX2OT mRNA level and decrease in the SOX2 mRNA level were noted, with impaired cognitive function, in the mice 3 days after tibial surgery compared with mice in the sham group. To evaluate the role of SOX2OT in PND, *SOX2OT* knockdown was performed in vitro and in vivo using lentivirus transfection in HT22 cells and via brain stereotactic injection of lentivirus, respectively. SOX2OT knockdown reduced apoptosis, inhibited oxidative stress, suppressed mitochondrial hyperdivision, attenuated surgery-induced cognitive dysfunction, and promoted downstream SOX2 expression in elderly mice. Furthermore, *Sox2* alleviated mitochondrial functional damage by inhibiting the transcription of mitochondrial division protein Drp1. Our study findings indicate that *SOX2OT* knockout alleviates surgery-induced mitochondrial fission and cognitive function defects by upregulating the expression of Sox2 in mice, resulting in the inhibition of drp1 transcription. Therefore, regulation of the SOX2/Drp1 pathway may be a potential mechanism for the treatment of patients with PND.

**Supplementary Information:**

The online version contains supplementary material available at 10.1186/s13041-023-01024-y.

## Introduction

Postoperative neurocognitive disorder (PND), the most common postoperative complication in elderly individuals, is mainly manifested as decline in learning and memory accompanied by executive, visual, and spatial and psychomotor functions. It can increase postoperative mortality rate, increase the risk of Alzheimer's disease in elderly patients, delay postoperative recovery, and reduce quality of life [1, 2]. Advanced age is considered a risk factor for the development of PND [3, 4], with 16–45% of elderly patients reportedly developing PND 3 months after joint arthroplasty [5]. However, the pathogenesis of PND remains unclear, and no effective treatments or preventive measures are available for patients with PND.

In mammals, mitochondria are important organelles and play a crucial role in energy metabolism and cell survival [6]. The high metabolic rate and the complex morphology of neurons render them particularly vulnerable to mitochondrial dysfunction [7, 8]. Mitochondria are dynamic organelles undergoing fission and fusion, wherein fission is mainly regulated by mitochondrial dynamin-related protein 1 (Drp1) and mitochondrial fission 1 protein (Fis1), and fusion is mainly regulated by the fusion proteins mitofusin 2 (Mfn2) and optic atrophy 1 (OPA1) [9, 10]. Changes in mitochondrial dynamics substantially affect mitochondria number and shape, ATP production, respiratory enzyme activity, and cell survival, as reported previously [11]. Considering the crucial role of mitochondria in neurons, dynamic mitochondrial damage is considered to be involved in neurodegenerative diseases [12–15]. Notably, the mechanisms and pathways through which mitochondrial defects lead to neuronal dysfunction in patients with PND are unclear.

Recently, a study has shown that long noncoding RNAs (lncRNAs) bind to other molecules or proteins and play a key role in brain neurotransmission, memory structure, and synaptic plasticity, as well as in the occurrence and regulation of neurodegenerative diseases [16]. The SOX2-overlapping transcript (*SOX2OT*) gene, which is located at chr3q26.33, is a highly conserved lncRNA in vertebrates. In an Alzheimer’s disease model, lncRNA *SOX2OT* transcription was accompanied by *SOX2* gene expression and associated with cognitive dysfunction [17, 18]. In a murine model of septic cardiomyopathy, *SOX2OT* knockout restored reduced cardiac function and improved mitochondrial membrane potential damage by lipopolysaccharides (LPS). Moreover, *SOX2OT* regulates mitochondrial dysfunction in septic cardiomyopathy through *SOX2* [19]. Here, we used lncRNA sequencing to evaluate the change in *SOX2OT* expression in a PND model. Furthermore, this study aimed to investigate the potential mechanism of *SOX2OT* in mitochondrial dynamics and cognitive impairment using a PND model. We hypothesized that *SOX2OT* knockdown could ameliorate surgery-induced cognitive dysfunction by upregulating *SOX2*.

## Methods

###  Animals

Male C57BL/6J mice (12 months old) weighing 30–35 g were purchased from the Air Force Military Medical University. All mice experiments were carried out in accordance with the guidelines for experimental animals of the National Institutes of Health and approved by the Animal Use and Care Committee of the Air Force Military Medical University (IACUC-20200650). All experimental mice were housed under a 12-/12-h light/dark cycle at 20–24 °C and 50–60% humidity with free access to food and water. In part I of the experiment, the mice were randomly divided into either a sham (n = 20) or surgery (n = 20) group.

In Part II, a mouse model with *SOX2OT* knockdown was established via stereotactic injection of lentivirus containing SOX2OT shRNA into the hippocampus. The tibial fracture model was established after a week, and the mice were divided into two groups: Lv-neg (stereotactic injection of empty lentivirus vectors; n = 10) and Lv-SOX2OT (stereotactic injection of Lv-SOX2OT; n = 10).

### Tibial fracture model

Tibial fracture surgery was performed, as reported previously [20–22]. Briefly, isoflurane (1.5–2.5%) and oxygen-mixed gas were administered via inhalation as anesthesia before tibial fracture surgery. Only the left hind limb was prepared for surgery. A 0.4-mm stainless steel needle was inserted into the intramedullary canal, the periosteum was separated, and osteotomy was performed. Finally, 0.2% ropivacaine was injected subcutaneously before closing the incision. The skin was then sutured with 4/0 nylon. Aseptic conditions were maintained throughout the procedure. The sham group was subjected to all surgical procedures except bone fracture and pin insertion.

### HT22 cell culture

HT22, a hippocampal neuronal cell line, was provided by Dr. Xiuquan Wu, from the Department of Neurosurgery, Air Force Military Medical University. All cells were cultured in complete medium at 37 °C in the presence of 5% CO_2_ under saturated humidity. HT22 cells were treated with lipopolysaccharide (LPS) to produce inflammatory factors to simulate the inflammatory reaction of the body. The cells were divided into four groups as follows: (1) the Lv-neg group (cells were transfected with empty lentivirus vectors for 48 h and replaced with fresh culture medium), (2) Lv-neg + LPS group (cells were treated with medium containing LPS [100 ng/mL] for 24 h after transfection of empty lentivirus vectors for 48 h), (3) Lv-SOX2OT group (cells were transfected with Sox2ot lentivirus for 48 h and then cultured in fresh medium for 24 h), and (4) Lv-SOX2OT + LPS group (cells were transfected with Sox2ot for 48 h and then treated with culture medium containing LPS [100 ng/mL] for 24 h).

### Apoptosis assay

According to a previous study [23], we used flow cytometry (Coulter XL, Beckman USA) to detect the apoptosis rate of HT22 cells with a PE Annexin V apoptosis detection kit (559763; BD Biosciences). Briefly, after being digested with trypsin, the cells were collected in a 15 mL tube and centrifuged at 900 r/min for 5 min and the supernatant discarded. The cells were then resuspended in 200 μL of combined buffer, collected in a flow tube, 5 μL of Annexin V PE was added into the flow tube, incubated at 24 °C for 10 min, and then washed once with 200 μL combined buffer. The cells were then centrifuged at 900 r/min for 5 min, and the supernatant was discarded. The cells were then resuspended with 190 μL of combined buffer, 10 μL of 7ADD was added into the flow tube, mixed, and subjected to flow cytometer. EXPO32-ADC, the system software of flow cytometry, was used for apoptosis analysis.

### Mitochondrial membrane potential analysis

Based on pre-established research methods [23] and the manufacturer's protocol, we used a JC-1 mitochondrial membrane potential determination kit (C2006; Beyotime Biotechnology, Shanghai, China) to measure the mitochondrial membrane potential.

### Detection of ROS in mitochondria and HT22 cells

Mitochondrial reactive oxygen species and total oxidative activity were analyzed using the mitochondrial fluorescent probe MitoSox (M36008; Thermo Fisher, USA) and a fluorescent intracellular reactive oxygen species kit (S0033; Beyotime Biotech, China), respectively, according to a previously reported methodology [24]. Images were acquired using a confocal laser-scanning microscope (FV 1000; Olympus, Japan).

### Estimation of mitochondrial morphology in HT22 cells

To observe the morphology of mitochondria, we used the MitoTracker probe (100 mM, 37 °C, 10 min) (M7510; Thermo Fisher Scientific, USA) to stain the cells. The image was then acquired using a confocal laser-scanning microscope (Olympus). We used Image-pro plus software to measure and analyze the number and volume of mitochondria per five separate regions of per sample, according to a previous study [25].

### Detection of mitochondrial oxygen consumption rate 

We used the XF24 extracellular flux analyzer (Agilent Seahorse Bioscience, USA) to determine the oxygen consumption rate (OCR) of HT22 cells [26]. The same numbers of HT22 cells (approximately 0.8 × 10^3^ cells per well) were cultured in Seahorse 24-well plates before OCR detection. HT22 cells were successively cultured in oligomycin to inhibit ATP synthase (1 μmol/L), carbonyl cyanide-p-trifluoromethoxyphenylhydrazone to promote the uncoupling of electron (FCCP; 0.5 μmol/L), and antimycin A to inhibitor the electron transfer (1 μmol/L). Thereafter, the baseline and maximum OCRs were calculated with these data. All OCR measurements were normalized to hemocytometry cell counts.

### RNA immunoprecipitation

All operations were conducted according to the instructions in the kit (#EZ-Magna RIP kit 17–701). Briefly, HT22 cells were collected and incubated with 5 µg of SOX2 (ab133337; Abcam)-specific antibody or normal IgG antibody for 2 h at 4 °C after adding an equal precipitation volume of RNA immunoprecipitation (RIP) complete lysate. The cell lysate was then added to the samples and spun overnight at 4 °C to produce beads, which were then washed six times with RIPA buffer. Finally, proteinase K treatment was used to release RNA from the bound protein. RNA was isolated using phenol:chloroform:isoamyl alcohol and precipitated using ethanol for the subsequent RT-PCR analysis.

### Luciferase reporter assay

To explore the SOX2-dependent transcription of Drp1, the 2 kb 5ʹ promoter (− 2000/0, see Additional file 1) sequence upstream of the mouse Drp1 transcription start site was cloned into a PGL3-basic vector upstream of the firefly luciferase gene. The plasmid was generated by Wuhan GeneCreate Biological Engineering Co., Ltd. Briefly, HEK293 cells were cultured and inoculated in 24-well plates. After 12 h of growth, the cells were transfected with either Drp1 promoter alone, co-transfected with the internal reference plasmid (*Renilla* luciferase reporter plasmid, pRL-TK), co-transfected with SOX2 small interfering RNA (siRNA), or empty virus for 24 h. After removing the transfection medium, the cells were supplemented with the medium containing 6 μM curcumin (Sigma-Aldrich, St. Louis, MO, USA), which was dissolved in 100% dimethyl sulfoxide. Forty-eight hours after transfection, the Dual-Luciferase Reporter Assay System (Promega) was used to measure luciferase activity. The pRL-TK vector was introduced to eliminate the intergroup error caused by cell transfection and other factors.

### Lentiviral infection

Lentiviral-SOX2OT is the SOX2OT silencing vector. The small volume infection method with a 1:2 ratio was used to replace the cell culture medium with serum-free culture medium, according to manufacturer’s instructions. After adding lentivirus infection to the serum-free medium for 4 h, the fresh medium was replenished. After continuously infecting the cells for 12 h, replace the fresh medium once, and then replace the fresh medium every 1–2 days according to the cell growth. After 48 h of transfection, cell fluorescence expression was observed, and the expression of LncRNA was detected using qRT-PCR.

### Western blotting

Mouse hippocampus tissue and HT22 cells were analyzed using western blotting, as described previously [27]. The cells were incubated overnight with the primary antibodies against Sox2 (ab171380, ab92494; Abcam), Drp1 (ab184248; Abcam), Fis1 (ab156865; Abcam), Opa1 (ab157457; Abcam), Mfn1 (ab221661; Abcam), Bcl-2 associated X (Bax, ab182733; Abcam), B-cell lymphoma-2 (Bcl-2, ab32124; Abcam), and β-actin (ab8227, Abcam) (all at 1:1000 dilution), followed by incubation with horseradish peroxidase-conjugated secondary antibodies (Santa Cruz, CA; 1:5000) at 24 °C for 2 h. The bicinchoninic acid (BCA) method was used to quantify the protein concentration, the value of each well was read by an enzyme-labeling instrument, and the concentration and sample loading amount of each group was calculated. Target proteins were visualized using chemiluminescence (Bio-Rad, Hercules, CA, USA). Band intensities were analyzed using ImageJ. β-actin was used as the loading control.

### Quantitative real-time PCR

RNA extraction and quantitative RT-PCR were performed according to the manufacturer’s protocol (Invitrogen, USA), as described previously [27]. PCR primers are summarized in Table 1. The final results were normalized to the expression levels of β-actin and expressed as fold change compared with the control.

**Table 1:** Primers used in qRT-PCR analysis

Gene	Forward (5ʹ)	Reverse (3ʹ)
*SOX2OT*	CAACTCGTTCTGTCCGGTGA	CCATGCCAGATCAGGGTGTT
*Sox2*	GGAAAGGGTTCTTGCTGGGT	ACGAAAACGGTCTTGCCAGT
*Dnajc19*	TGGAGTCATGGCCGTTATGT	AATGCTGCTTCCCGTTTTGT
*Fxr1*	CACGAAGACTCCCTCACAGT	CATCCGAACTTTAGCCAGCC
*β-actin*	GATCCATGAGACCACCTACAAC	TCAGCGATACCAGGGTACAT

### Neurobehavioral tests

Open field (OFT), novel object recognition (NOR), and fear conditioning (FCT) tests were performed on day 3 post-operation. The same behavioral tests were used for both the sham and surgery groups.

OFT was conducted to evaluate anxiety and locomotor activity in the experimental mice [28]. Mice from the sham and surgery groups were placed in a 40 cm × 40 cm × 40 cm plastic box and allowed to move freely in the box for 5 min before testing. The total distance traveled and amount of time spent by each mouse in the central square were recorded by an observer who was blinded to animal grouping.

NOR was used to test the recognition and nonspatial memory abilities of the mice [29]. Briefly, the mice were allowed to explore two identical objects (A and B) that were placed 10 cm away from the wall of the box during training. The recognition test phase was performed 24 h after the end of the familiarization phase. Object B was replaced with a new object C, and the mice were allowed to explore freely for 5 min. The duration of exploration for each object was recorded. The total sniffing time of novel object was recorded for further analyses. The new object preference index was calculated as follows:

$$T_{{\text{C}}} /\left( {T_{{\text{A}}} + T_{{\text{C}}} } \right) \, \times { 1}00\% ,$$

where *T*_A_ and *T*_C_ are the time spent exploring objects A and C, respectively.

When experiencing an aversive stimulus, mice adopt a typical “freezing” posture, which is memorized via contextual clues that are related to a previously learned fear-inducing stimulus–response pairing [30]. In this study, sound was used as a conditioning stimulus, and an electric shock to the foot acted as an unconditional stimulus. Sound (2000 Hz and 90 db for 30 s) and electrical (1 mA for 2 s) stimuli administered during the training phase indicated that the mice would associate the link between the environment and electric shock following training. A contextual test was performed in a similar context chamber for 5 min without any sound or electric shock stimulation. During the cued test, the cue (90 db sound stimuli) was applied in a plastic box with black and white stripes different from those of the pre-operation training. The cued test was performed after completion of the contextual test.

After the behavioral test, the mice were euthanized, their brain tissue was removed immediately and placed on ice, and the hippocampal tissue was isolated and cryopreserved at − 80 °C for lncRNA sequencing (Shanghai Jingneng Company).

### Cell Counting Kit-8 (CCK-8) and lactate dehydrogenase analysis

CCK-8 (Dojindo ck04) and lactate dehydrogenase (LDH) (c0017; Beyotime) analyses were performed after the cells were subjected to lentiviral infection. Cell viability was analyzed using the CCK-8 kit according to the manufacturer’s instructions. After incubating the cells for 1–4 h, color changes were observed, the absorbance at 450 nm was measured using a microplate reader. The cell survival rate was calculated according to the absorbance value [31]. Prism 8.0 software was used to analyze data. The amount of LDH released in each group was measured following the instructions on the LDH assay kit. The cells were divided into different groups and then cultured again. LDH release was detected at 24, 48, and 72 h. the absorbance at 490 nm was measured using a microplate reader. LDH enzyme activity was calculate according to the absorbance value [32]. The time at which neuronal injury occurred was determined for the subsequent experiment.

### Transmission electron microscopy (TEM)

According to previous research [33], the hippocampus sample from the brain was obtained, fixed with 2.5% glutaraldehyde (pH 7.4, 4 °C) for 24 h, and then fixed with 1% osmium tetroxide in deionized water for 4 h. All sections were observed using a TEM (JEM-1230; JEOL Co., Ltd., Tokyo, Japan). We generated a semi-quantitative score for the morphological damage of mitochondria [34]: 0 = no damage, normal morphology, electronic dense structure; 1 = mild injury: the number of cristae decreased, slightly swollen, and the electronic dense appearance disappeared; 2 = severe injury: (near) the cristae completely disappeared, and the mitochondria were severely swollen; 3 = complete destruction: outer membrane rupture, mitochondrial structure collapse. Image J software was used to score all mitochondria in five separate regions of each sample, and the average score of each region was plotted.

### Small interfering RNA (siRNA) transfection 

SOX2 siRNA was provided by Hanbio Company, Shanghai, China. Diluted siRNA and RNA transfection partners (Lipofectamine; Hanbio Company Shanghai, China) were added to six-well (1500 µL/well) plates when HT22 cells reached 60–70% confluency, and siRNA transfection was performed in Opti-MEM medium. After 4 h, fresh medium was added to each well. Transfection efficiency was evaluated by measuring the expression level of SOX2 protein.

### Lentivirus stereotactic injection

Mice were anesthetized via the intraperitoneal injection of ketamine (0.1 mg/kg) before the skin was prepared and fixed on the brain stereotactic injection instrument to ensure the symmetrical placement of ear rods. After complete disinfection, the skin of the skull top was cut open on both sides and the bregma and lambda points were fully exposed on the skull top plane. Craniotomies were performed at ± 1.85 mm anterior to the bregma and − 1.94 mm lateral to the midline. Coordinates were determined according to the mouse brain atlas (x =  ± 1.85 mm, y = − 1.94 mm, z = − 2 mm) and holes were drilled into the skull with a dental drill. Two sites in the CA1 region of the hippocampus were selected for injecting the virus, and 1 µL of virus was injected to each site over 30 min. The needle was left in place for 10 min. After the localization injection, the skin on top of the skull was disinfected and sutured. The mice were returned to the cage to continue feeding when fully awake.

### LncRNA sequencing

LncRNA sequencing analyses were conducted to compare the hippocampal tissues of mice from the PND and control groups. Shanghai Jingneng Co., Ltd. Gene Ontology (GO) analysis was used to analyze the main functional differentially expressed genes, and Kyoto Encyclopedia of Genes and Genomes (KEGG) analysis was used to analyze the signal pathway of differential gene enrichment (P < 0.05) (see Additional file 2).

### Statistical analysis

All values are presented as mean ± standard error. T-test was used for the analysis of two groups of data, multiple groups of data analysis was performed using ANOVA followed by Tukey's post-hoc test with GraphPad Prism 8.0. Statistical significance was set at P < 0.05.

## Results

### Cognitive impairment in a mouse model of tibial fracture

**Fig. 1:**
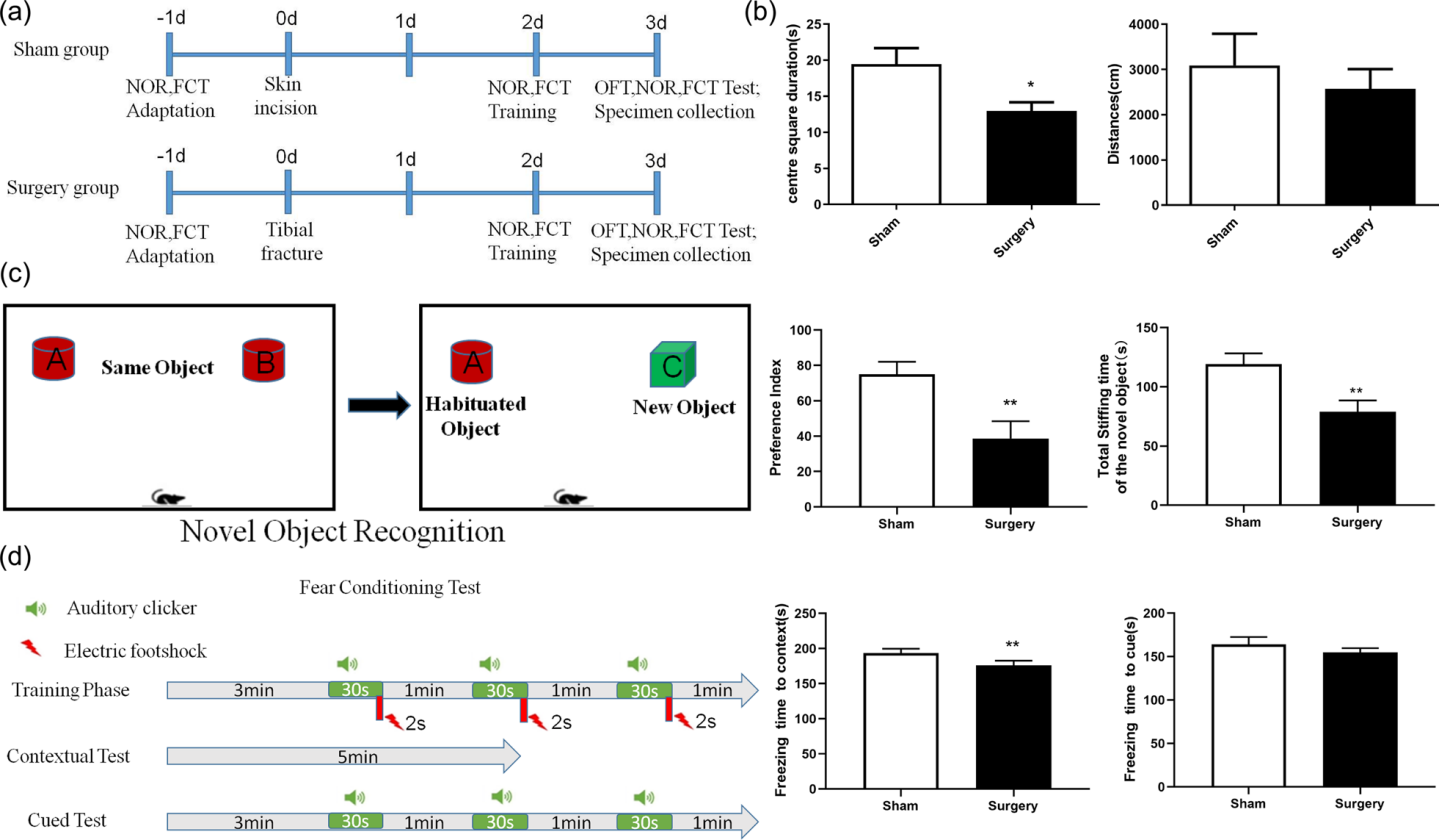
Experimental design and behavioral test results. **a** Mice were provided behavioral training 1 day before tibial surgery. The sham group mice were not subjected to tibial fracture. Behavioral tests were performed on day 3 post-surgery. **b** Comparison of the total activity, distance, and time spent in the central square. *P* < 0.05 vs. sham group; n = 4. **c** Comparison of total sniffing time and preference index for novel object. **P < 0.001 vs. sham group; n = 4. **d** Freezing time. **P < 0.005 vs. sham group; n = 4. Red arrows indicate electric shock stimuli, and green horn indicates sound stimuli

Figure 1a shows the neurobehavioral test design. In the OFT, no significant differences were noted in locomotory activity between the sham and surgery groups. The mice in the surgery group spent less time in the center square than those in the sham group (Fig. 1b), indicating no significant difference in exercise ability between the groups. The total sniffing time of novel objects was significantly lower in the surgery mice group than in the sham group. In addition, mice in the surgery group had a lower preference index than those in the sham group (*P* < 0.001; Fig. 1c). These results suggest that mice in the surgery group failed to recognize the difference between novel and familiar objects. The fear conditioning (FCT) workflow is shown in Fig. 1b. In the contextual test, the freezing times for mice in the surgery group were less than those for mice in the sham mice (*P* < 0.05; Fig. 1d); however, no significant difference was noted in freezing time between the groups in the cued test (Fig. 1d).

### Involvement of differentially expressed lncRNA *SOX2OT* in mitochondrial dysfunction

**Fig. 2:**
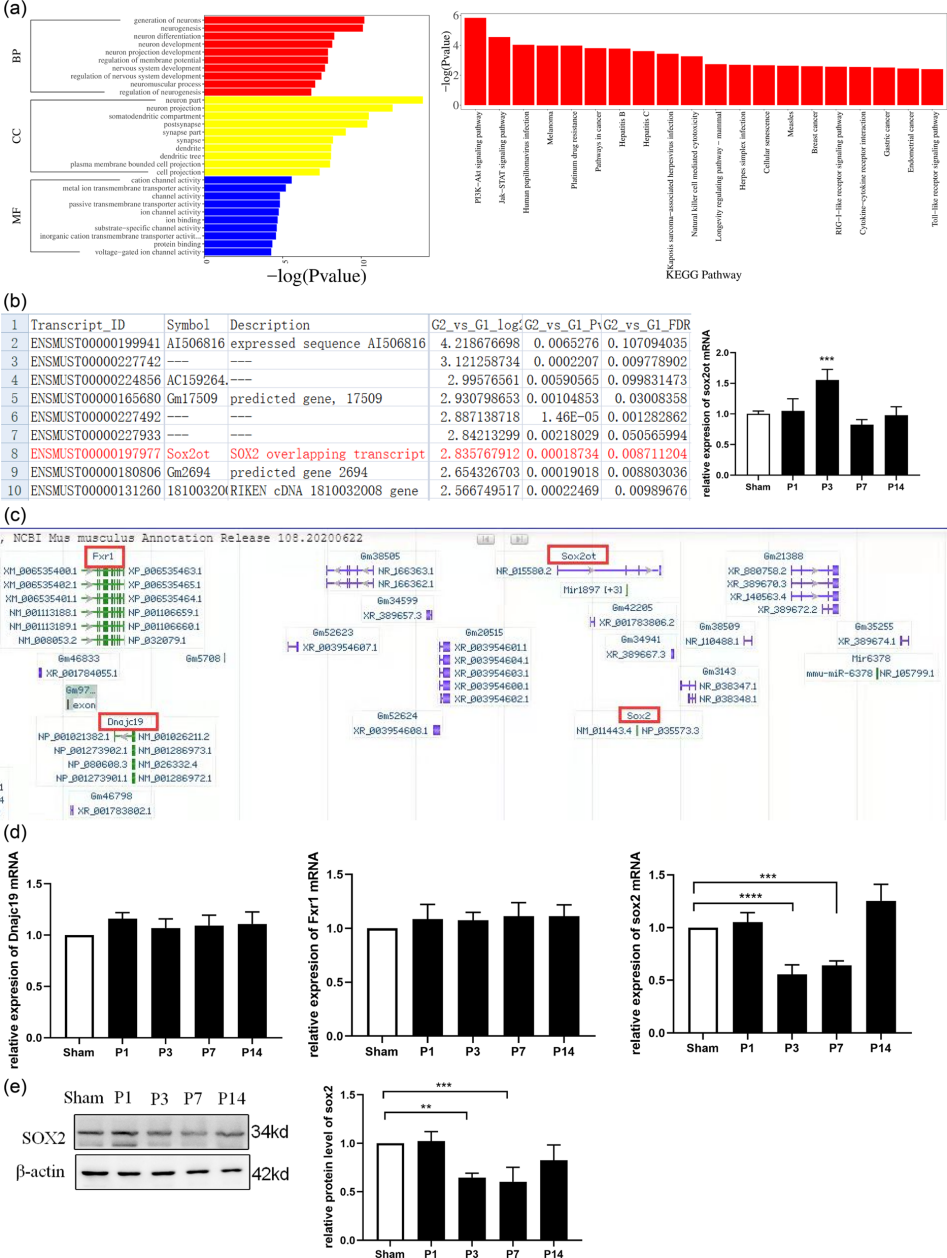
Upregulated lncRNA *SOX2OT* expression in the mouse model of tibial fracture. **a** GO term and KEGG pathway enrichment map of differential lncRNAs. **b** High expression of *SOX2OT* in lncRNA sequencing, verified using RT-PCR (*P* < 0.0001). **c** *SOX2OT* located on mouse chromosome 3 and *Fxr1*, *Sox2*, and *Dnajc19* are upstream and downstream genes of *SOX2OT* in the Ensembl database. **d** *Dnajc19, SOX2*, and *Fxr1* mRNA levels measured using RT-qPCR on days 1, 3, 7, and 14 after sham treatment or surgery. *P < 0.05, **P < 0.002 vs. sham group. **e** Western blot analysis showing SOX2 protein expression on days 1, 3, 7, and 14 day after sham treatment or surgery. *P < 0.05 vs. sham group; n = 6

The KEGG pathway analysis indicated that differentially expressed lncRNAs were mainly involved in PI3K-Akt and JAK-STAT signaling (Fig. 2a). lncRNA sequencing showed that *SOX2OT* was upregulated in the PND model (*P* < 0.0001; Fig. 2b). We used lncRNA sequencing and the Ensembl database to predict that the *cis*-regulated target genes for lncRNA *SOX2OT* were *SOX2*, *DNAJC19*, and *FXR1* (Fig. 2c). Verification of these results using RT-PCR showed that the mRNA expression of *SOX2* decreased 3 days after surgery (*P* < 0.002), whereas no significant changes were observed in *DNAJC19* and *FXR1* mRNA levels (Fig. 2d). Western blotting confirmed that SOX2 protein expression significantly decreased 3 days after surgery (Fig. 2e).

### Knockdown of *SOX2OT* inhibited apoptosis

**Fig. 3:**
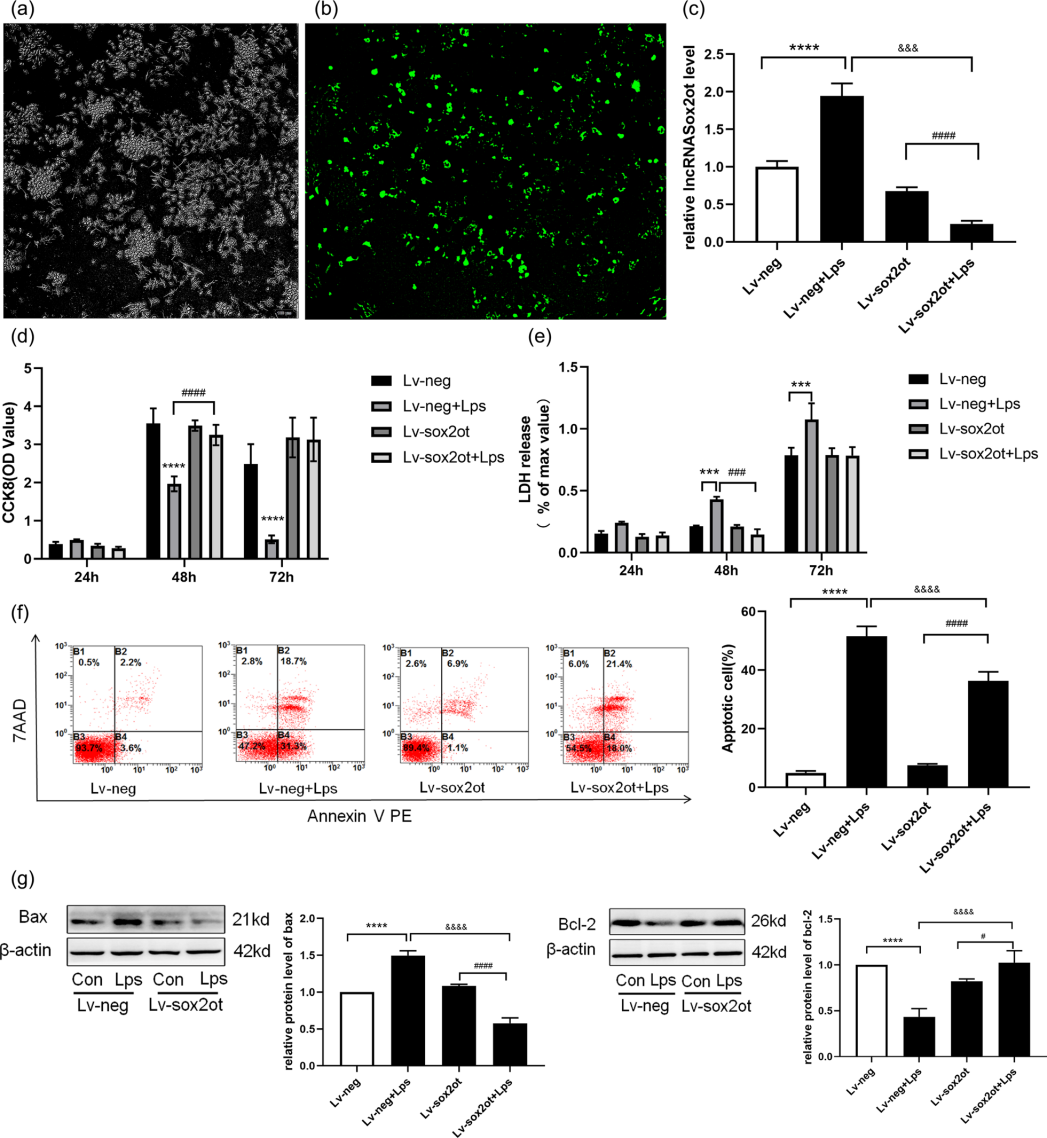
*SOX2OT* knockdown suppresses apoptosis. **a–c** Fluorescent images showing virus-transfected HT22 cells and the stable knockdown of *SOX2OT*; SOX2OT expression levels detected using RT-PCR in the Con and LPS groups. *P < 0.05, ****P < 0.0001 vs. Lv-neg; ^####^*P* < 0.0001 vs. Lv-neg + LPS. **d** HT22 Lv-neg or Lv-SOX2OT cells were treated with LPS (100 ng/mL) for 24, 48, and 72 h, and then cell viability was determined. *P < 0.05 vs. Lv-neg; ^#^*P* < 0.05 vs. Lv-neg + LPS. **e** Activity of lactate dehydrogenase in HT22 Lv-neg or Lv-SOX2OT cells cultured with LPS (100 ng/mL) for 24, 48, and 72 h using the LDH kit. *P < 0.05, ***P < 0.0003 vs. Lv-neg;^####^*P* < 0.0001 vs. Lv-neg + LPS. **f** Apoptosis detected using flow cytometry. ****P < 0.0001 vs. LV-neg; ^####^*P* < 0.0001 vs. Lv-SOX2OT + LPS; ^&&&&^*P* < 0.0001 vs. Lv-neg. **g** Western blot analysis showing Bax and Bcl-2 protein expression after transfection with Lv-SOX2OT or Lv-neg lentiviral vectors. ****P < 0.0001 vs. Lv-neg; ^####^*P* < 0.0001 vs. Lv-SOX2OT + LPS; ^&&&&^*P* < 0.0001 vs. Lv-neg; n = 4

HT22 cells were transfected with lentiviral vectors to knockdown *SOX2OT*. The knockdown efficiency was confirmed using fluorescence microscopy and qRT-PCR analysis (Fig. 3a–c). The cell counting kit-8 (CCK-8) assay showed that *SOX2OT* knockdown significantly increased cell proliferation and viability in the Lv-neg group (Fig. 3d), and the lactate dehydrogenase (LDH) assay showed that Lv-SOX2OT-transfected HT22 cells released less LDH (*P* < 0.05) (Fig. 3e). Furthermore, examination of HT22 apoptosis using flow cytometry and western blotting revealed that the loss of *SOX2OT* caused a significant decline in apoptosis compared with that observed in Lv-neg HT22 cells (Fig. 3f). In addition, a positive correlation was found between SOX2OT and Bax, whereas a negative correlation was found between SOX2OT and Bcl-2. *SOX2OT* knockdown increased Bcl-2 protein expression and reduced Bax protein expression (Fig. 3g). These results suggest that silencing SOX2OT expression inhibits apoptosis.

### Silencing *SOX2OT* expression reduced mitochondrial fission

**Fig. 4:**
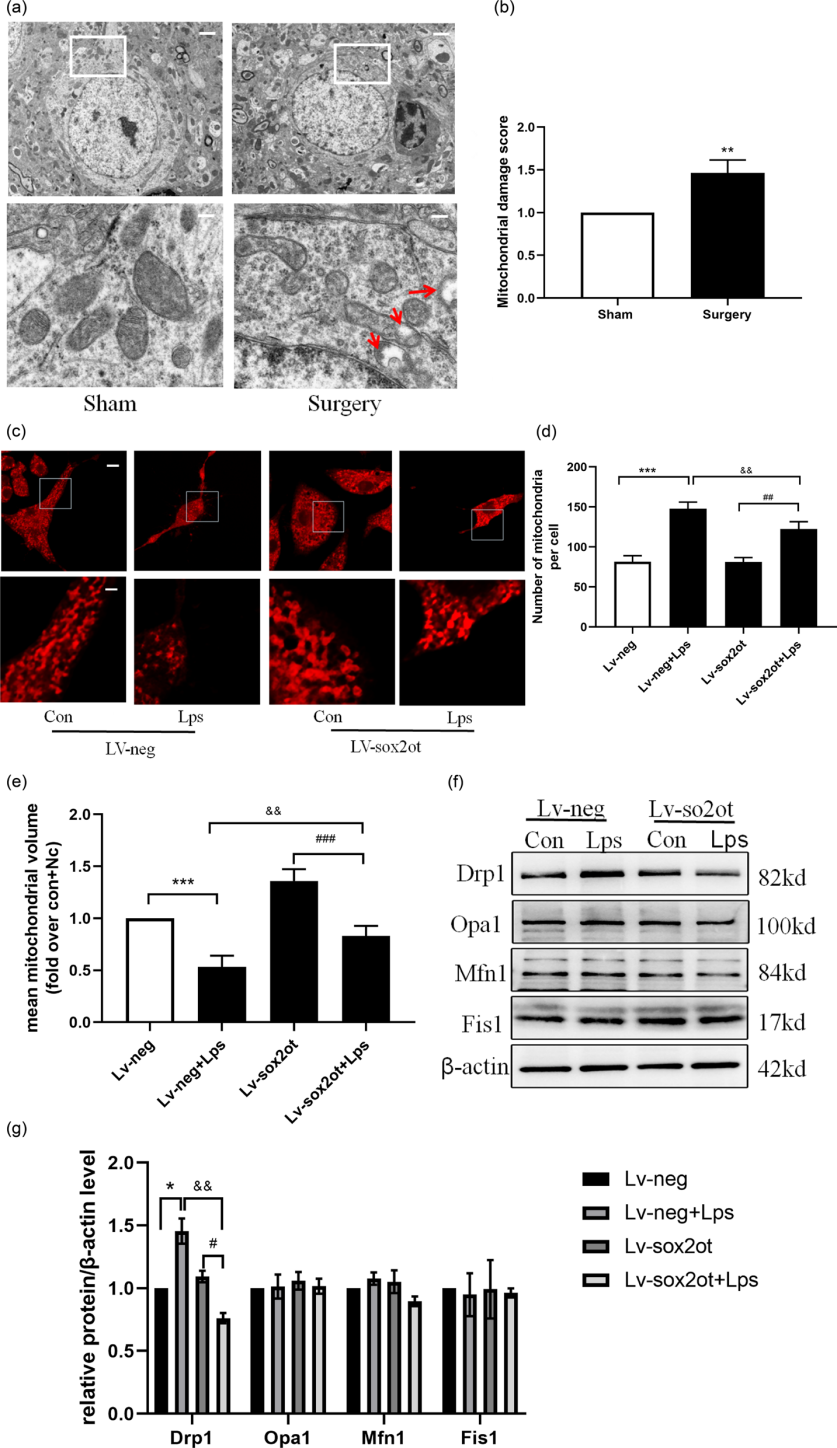
*SOX2OT* knockdown prevents LPS-induced mitochondrial fission. **a** Mitochondrial morphology in the hippocampus determined using transmission electron microscopy. Scale bar = 0.5 μm. Analysis of mitochondrial damage score. *P* < 0.005 vs Sham. **b** Mitochondrial morphology in HT22 cells obtained using MitoTracker Red staining. Original magnification × 600; n = 4. **c** Percentage of cells with fragmented mitochondria. **d:** Mean mitochondrial volume (fold over Con + Nc); n = 4. **e** Expression of the Drp1, Fis1, Mfn, and Opa1 proteins determined using western blotting. *P < 0.05, ****P < 0.0001 vs. Lv-neg; ^##^*P* < 0.01 vs. Lv-neg + LPS; ^&&^*P* < 0.009 vs. Lv-SOX2OT groups; n = 4

Electron microscopy showed that the ultrastructure of neurons in the operation group changed significantly; specifically, the mitochondria swelled significantly, and the mitochondrial crista broke, indicating vesicular changes. Further analysis of the mitochondrial damage score showed that the mitochondrial damage score of the surgical group was significantly higher than that of the sham group (Fig. 4a). The MitoTracker Red probe was used to examine mitochondrial morphology in HT22 cells. Mitochondria in normal HT22 cells mainly presented as long tubules with interconnecting networks (Fig. 4b). After 24 h of LPS stimulation, the mitochondria of HT22 cells became shorter and smaller, with more fragments. However, *SOX2OT* knockdown prevented mitochondrial fission in HT22 cells compared with that in Lv-con HT22 cells (Fig. 4c, d). Additionally, the expression levels of the major mitochondrial fission (Drp1 and Fis1) and fusion (Opa1 and Mfn1) proteins were evaluated. Lv-neg + LPS cells showed higher Drp1 levels than Lv-negHT22 cells, whereas the expression levels of Fis1 and the fusion proteins remained unchanged (Fig. 4e). These results suggest that the mitochondrial fission mediated by Drp1 is enhanced in the LPS-induced inflammatory responses. However, Drp1 expression was reduced following *SOX2OT* knockdown (Fig. 4d), suggesting that silencing *SOX2OT* inhibits mitochondrial hyperfission.

### Silencing *SOX2OT* expression protected against LPS-induced oxidative stress and mitochondrial dysfunction

**Fig. 5:**
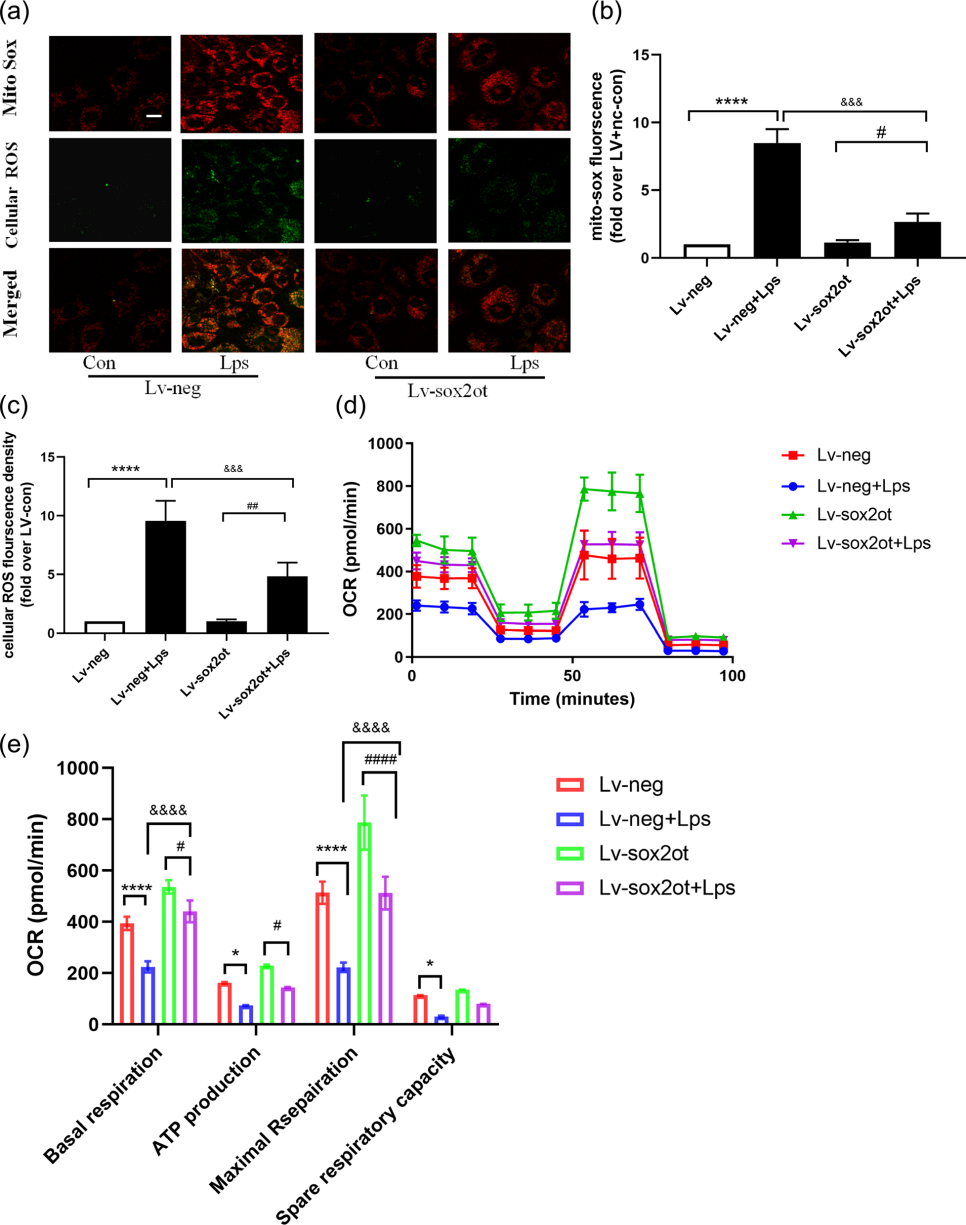
*SOX2OT* knockdown protected against LPS-induced mitochondrial dysfunction in HT22 cells.** a** Mitochondria-derived superoxide was stained with the MitoSox probe, and intracellular oxidants were stained with cellular ROS dye. Original magnification × 600; n = 4. **b**, **c** Statistical analysis of cellular ROS fluorescence density and MitoSox fluorescence in HT22 cells (fold over Lv + nc); n = 4. **d–e** Seahorse analysis showing OCR and quantitative statistical analysis of OCR. *P < 0.05 vs. Lv-neg; **P < 0.01, ****P < 0.01 vs. Lv-neg; ^##^*P* < 0.01 vs. Lv-neg; ^&&&^*P* < 0.01 vs. Lv-neg + LPS; n = 5

LPS-cultured HT22 cells showed increased intracellular ROS (indicated in green) and mitochondrial ROS (indicated in red, Fig. 5a–c) levels. The results of co-staining (indicated in yellow) showed that ROS mainly originated from the mitochondria. *SOX2OT* knockdown effectively reduced oxidative stress and mitochondria in LPS-treated cells. The respiratory capacity of mitochondria was examined using a Seahorse cell capacity analyzer. LPS treatment significantly decreased basal respiration, ATP production, and maximal respiration of mitochondria in HT22 cells, and these effects were significantly ameliorated in *SOX2OT* knockdown HT22 cells (Fig. 5d–e). These findings suggest that *SOX2OT* knockdown alleviates LPS-induced oxidative stress and mitochondrial dysfunction.

### *SOX2OT* knockdown attenuated surgery-induced cognitive dysfunction and promoted downstream SOX2 expression in elderly mice

**Fig. 6:**
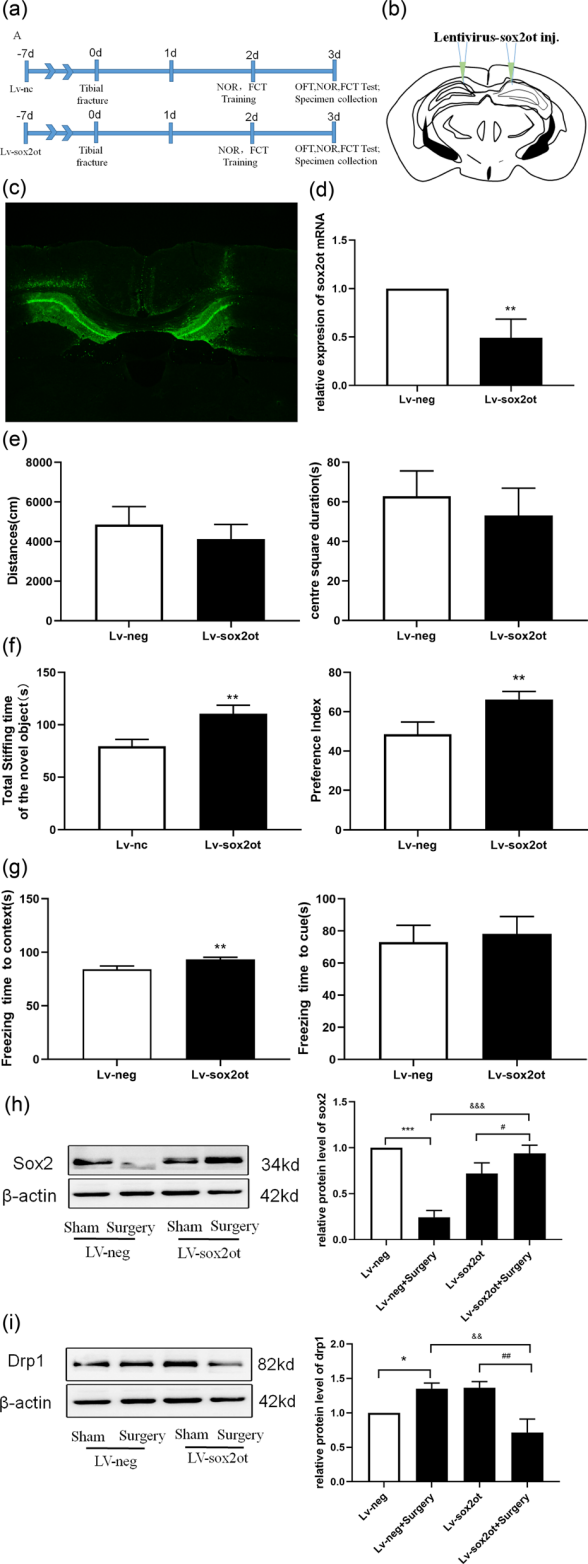
*SOX2OT* knockdown attenuated surgery-induced cognitive dysfunction and promoted downstream *SOX2* expression in elderly mice. **a** Experimental design and workflow. **b** The lentivirus Sox2ot was injected bilaterally into the CA1 region of the mouse hippocampus. **c** The GFP signal observed in CA1 neurons 7 days after injection of lentivirus confirmed the successful expression of Sox2ot or the control vector. Scale, 100 μm. **d** SOX2OT mRNA measured using RT-qPCR. **P < 0.001 vs Lv-neg. **e** Comparison of total activity, distance, and time spent in the central square; n = 4. **f** Comparison of total sniffing time and preference index for novel object. **P < 0.001 vs. Lv-neg group; n = 4. **g** Freezing time. **P < 0.005 vs. Lv-neg. **e** SOX2OT mRNA measured using RT-qPCR. **P < 0.001 vs Lv-neg. **h** SOX2 protein expression analyzed using western blotting. ***P < 0.0001, ^#^*P* < 0.03, ^&&&^*P* < 0.0001 vs Lv-neg. **i** Drp1 protein expression analyzed using western blotting. *P < 0.04, ^###^*P* < 0.0006, ^&&^*P* < 0.003 vs Lv-neg; n = 4

To clarify the role of *SOX2OT *in vivo, lentivirus was injected into the brain of aged mice to interfere with *SOX2OT* expression. Figure 6a is an experimental flow chart that describes the process followed. Seven days after lentivirus injection, several CA1 pyramidal neurons were completely infected successfully. Compared with the Lv-neg group, the expression of Sox2ot mRNA in the Lv-SOX2OT group decreased significantly (p < 0.05) (Fig. 6b–d). No significant differences were observed in the total activity distance and center square duration between the groups in the OFT, as shown in Fig. 6e. However, both the total stiffing time of novel object and preference index were significantly higher in the Lv-SOX2OT group than in the Lv-neg group (Fig. 6f) in the mice NOR experiment. Mice in the Lv-SOX2OT group demonstrated longer freezing times in the Contextual Fear Conditioning Test (FCT) compared to those in the Lv-neg group, as shown in Fig. 6g; however, no significant difference was noted in the freezing time between the groups in the cued FCT. These results suggest that silencing *SOX2OT* expression protects against context-dependent dysfunction. Western blotting results showed that the level of SOX2 expressed by mice in the Lv-neg + Surgery group was lower than that in the Lv-neg group, and the opposite effect was observed in the Lv-SOX2OT group (Fig. 6h). In addition, the level of Drp1 protein in the Lv-neg + Surgery group mice was higher than that in Lv-neg group mice. However, Lv-SOX2OT mice showed the opposite results (Fig. 6i). These results suggest that *SOX2OT* knockdown reduces excessive mitochondrial division caused by surgery.

### *SOX2OT* bound to the downstream *SOX2* target, which was related to the mitochondrial fission protein Drp1

**Fig. 7:**
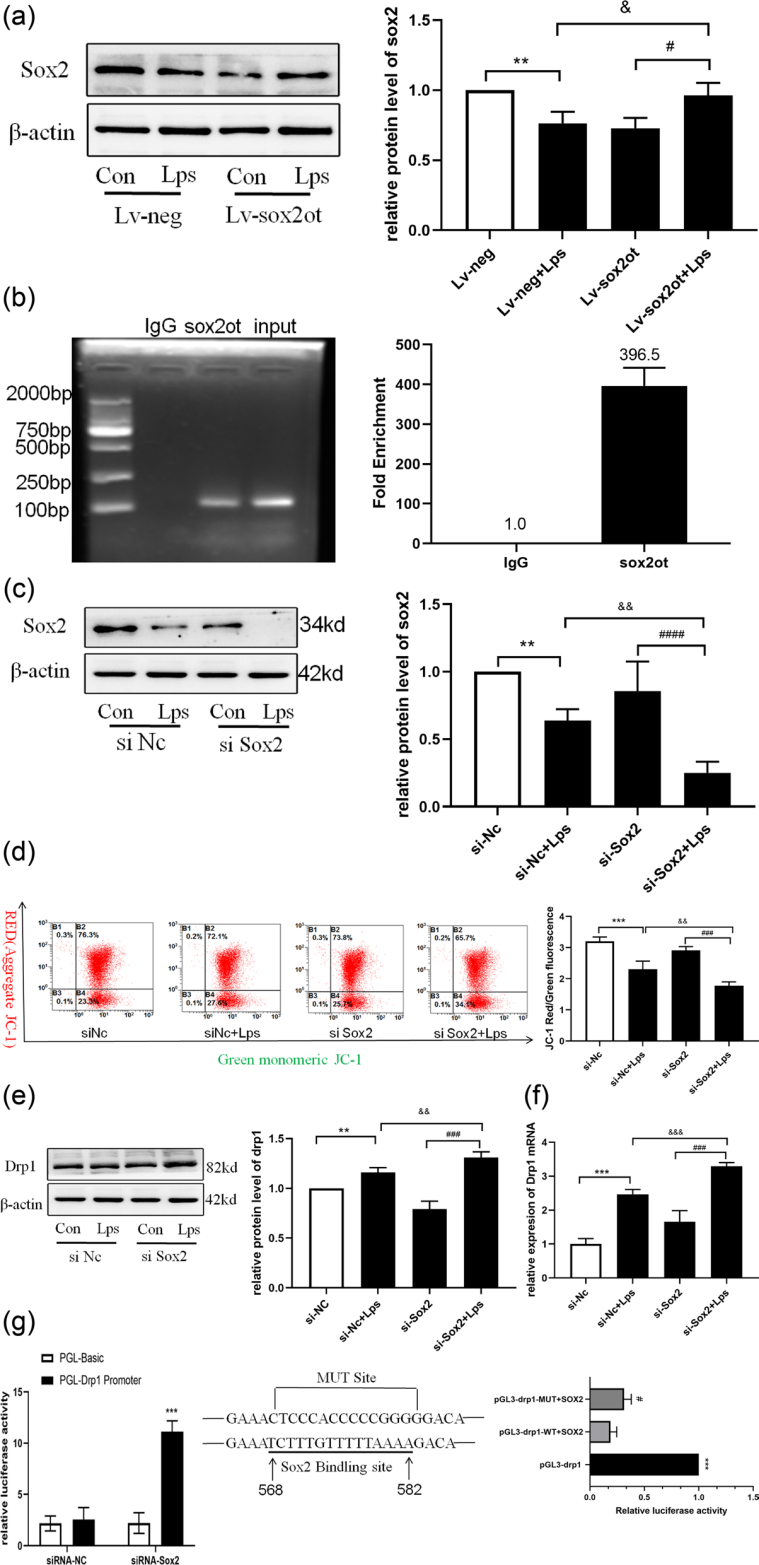
*SOX2OT* binds to the downstream *SOX2* target, which is related to mitochondrial fission protein Drp1. **a** Evaluating the transfection efficiency of Lv-SOX2OT lentivirus by detecting the expression of SOX2 using western blotting. *P < 0.05 vs. Lv-neg group, ^#^*P* < 0.05 vs. Lv-SOX2OT group, ^&&^ *P* < 0.004 vs. Lv-neg + LPS group; n = 4. **b** RIP-qPCR to verify the binding of downstream SOX2 target with lncRNA SOX2OT. *P < 0.05 vs. IgG group; n = 3. **c** Verifying the transfection effect of siRNA by detecting the expression of SOX2 using western blotting. **P < 0.007 vs. si-nc group, ^####^ *P* < 0.0001 vs. si-SOX2 group; ^&&^*P* < 0.004 vs. si-nc + LPS group; n = 4. **d** Flow cytometry analysis and JC-1 quantitative determination of mitochondrial membrane potential in HT22 cells. ****P < 0.0001 vs. si-nc group; ^###^*P* < 0.001 vs. si-SOX2 group; ^&&^*P* < 0.004 vs. si-nc + LPS group; n = 4. **e** Drp1 expression detected using western blotting after transfection with si-NC or si-SOX2 vectors. **P < 0.006 vs. si-nc group; ^###^*P* < 0.0001 vs. si-SOX2 group; ^&&^*P* < 0.009 vs. si-nc + LPS group; n = 4. **f **The drp1 mRNA level measured using RT-qPCR.^***^P<0.001 vs. si-NC; ^&&&^*P*<0.001 vs. si-Nc+LPS; ^###^*P*<0.001 vs. si-SOX2. **g** Analysis of the binding reactivity between SOX2 and drp1 promoter region using Luciferase reporter assay. ***P < 0.0006 vs.pGL3-*drp1* ^#^*P* < 0.05 vs. pGL3-*drp1-*WT + SOX2; n = 3

We confirmed that SOX2 gene and protein expression decreased in mice with postoperative tibial fractures (Fig. 2d, e). Similarly, the expression of SOX2 decreased when HT22 cells were treated with LPS, which is consistent with the expression trend in postoperative mouse brain tissue. Finally, *SOX2OT* knockdown led to an increase in SOX2 expression (Fig. 7a), indicating that *SOX2OT* is negatively correlated with *SOX2* expression. These findings suggest that SOX2 acts as a target gene of SOX2OT in PND mice.

*SOX2OT* negatively regulates the self-renewal capability of neural stem cells, is mainly expressed in the nucleus, and suppresses the level of SOX2. The transcription factor SOX2 directly binds to the* SOX2OT* promoter to promote its transcription [35]. To verify whether there is interaction between SOX2OT and SOX2 in this experiment, we used RIP-qPCR to show that SOX2 could combine with SOX2OT (Fig. 7b). Silencing *SOX2* expression with siRNA significantly decreased the mitochondrial membrane potential (Fig. 7c, d).

**Fig. 8:**
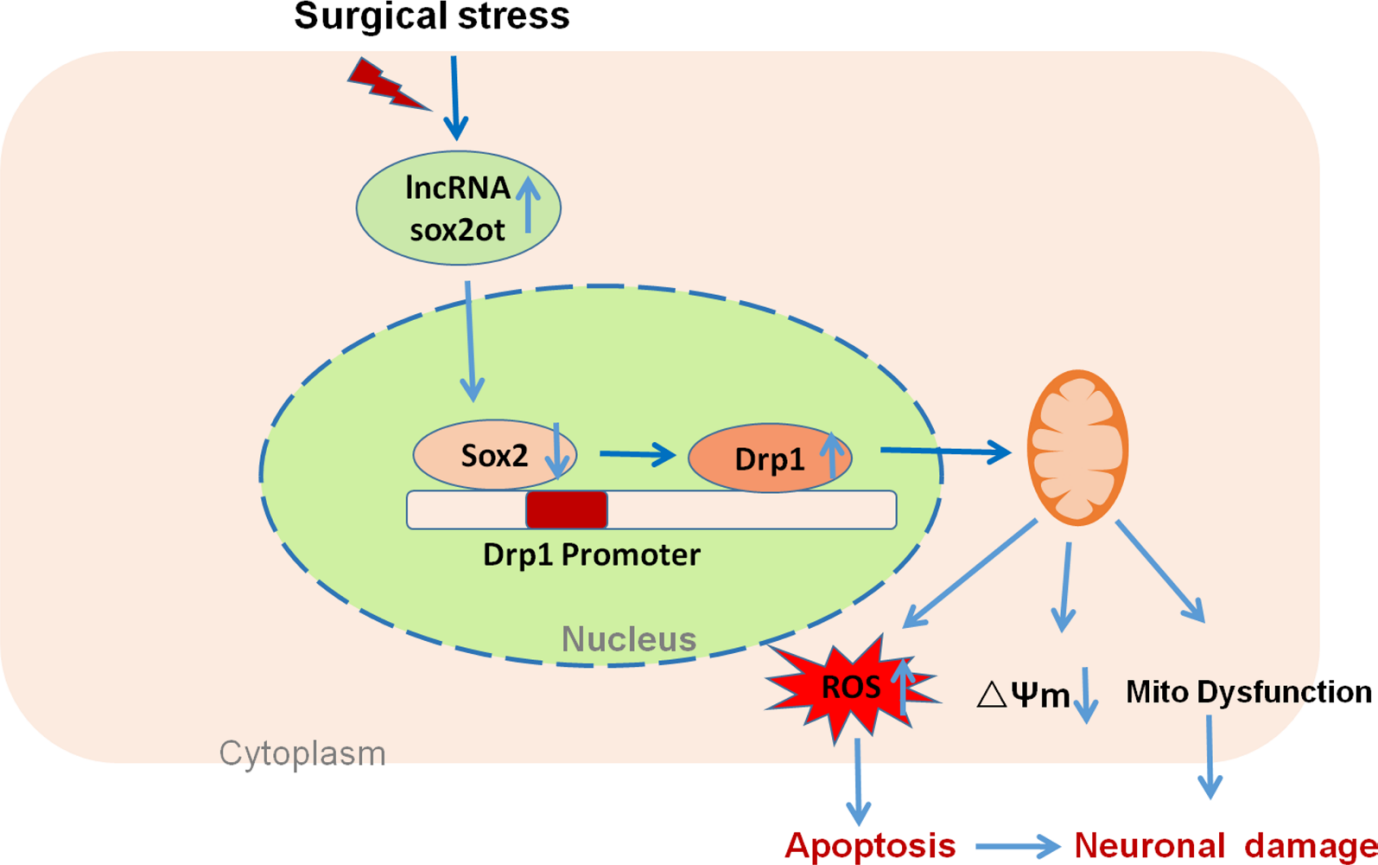
Surgical stress leads to mitochondrial dynamics imbalance due to increased *SOX2OT* expression, promoting neuronal damage. *SOX2OT* negatively regulates SOX2 expression, which negatively regulates Drp1 expression. Surgical stress increases *SOX2OT* and Drp1 expression, leading to oxidative stress and mitochondrial-derived ROS production. Thus, the mitochondrial membrane potential is reduced and mitochondrial dysfunction occurs, leading to the development of postoperative cognitive dysfunction in elderly mice. *SOX2OT* knockdown can reduce oxidative stress and mitochondrial dysfunction, alleviate apoptosis, and protect against inflammation-induced cognitive dysfunction

Interestingly, compared with the si Sox2 group, the si SOX2 + LPS group showed a significant increase in the expression of the fission protein Drp1 (Fig. 7e–f). To explore the mechanism by which SOX2 regulates Drp1 expression, a Drp1 promoter was cloned upstream in the pGL3.0 Basic plasmid for the luciferase reporter assay. The Drp1 promoter, which is involved in SOX2 responsiveness, was present in the 568–582 bp range (TCTTTGTTTTTAAAA). When SOX2 binds to the binding site of *drp1*, the luciferase activity suddenly decreases, making the luciferase activity increase after the binding site mutation (Fig. 7g). These results indicate that SOX2 combines with the Drp1 promoter sequence to affect its expression. Collectively, the findings of this study suggest that the SOX2OT–SOX2 signaling pathway affects postoperative cognitive function through Drp1-mediated mitochondrial dynamics (Fig. 8).

## Discussion

In clinical practice, the incidence of PND in patients undergoing orthopedic surgery is higher in elderly patients that in young patients. Increasing evidence suggests that mitochondrial disorders play a major role in postoperative neurocognitive dysfunction [36–38]. However, the underlying pathological mechanisms of postoperative neurocognitive dysfunction remain unclear. In this study, we made several important observations regarding PND pathogenesis. First, tibial surgery leads to both cognitive dysfunction and *SOX2OT* upregulation. Second, *SOX2OT* knockdown reduces apoptosis, inhibits oxidative stress, and improves mitochondrial function. Third, *SOX2OT* knockdown reduces surgery-induced cognitive dysfunction and promotes downstream *SOX2* expression in elderly mice. Finally, *SOX2OT* knockdown reduces oxidative stress and mitochondrial dysfunction by increasing SOX2 expression, which is related to the mitochondrial fission protein Drp1. These results show that SOX2OT inhibition can protect the brain from surgery-induced mitochondrial defects and cognitive dysfunction.

In this study, we observed a decline of cognitive function three days after the operation, mainly in the mice NOR test, and a significant reduction both in the total sniffing time and preference for new objects. The freezing time for fracture-induced mice significantly shortened in FCTs, indicating that cognitive dysfunction occurred in mice after surgery. The cognitive function of mice was improved by stereotactic injection of a lentivirus to knockdown SOX2OT expression. In recent years, the lncRNA *SOX2OT* has gained attention for its impact on mitochondrial function in the central nervous system [39, 19]. A recent study reported that, in a mice model of septic cardiomyopathy, the overexpression of SOX2OT leads to mitochondrial dysfunction [35]. In addition, in a sepsis model, the activation of SOX2OT in the mouse hippocampus led to defects in hippocampal neurogenesis and cognitive function, whereas the knockdown of SOX2OT alleviated these symptoms. In contrast, *SOX2OT* silencing attenuated neurogenesis impairment and cognitive dysfunction caused by sepsis [40]. In this study, we found that surgery promoted *SOX2OT* expression in the brain tissue and that *SOX2OT* silencing alleviated mitochondrial and cognitive function deficits associated with surgery. Our results are consistent with those of previous research, which associated *SOX2OT* overexpression with neurocognitive dysfunction in Alzheimer’s disease [17].

Neuronal mitochondrial dynamics are essential for neuronal development, plasticity, and functions [41, 42]. Mitochondria maintain a dynamic balance of fusion and fission. Abnormal mitochondrial fusion and fission can block the transport of mitochondria in neurons, disrupt their distribution, reduce energy efficiency, and increase ROS production, leading to synaptic dysfunction and nerve cell death [43, 44]. Moreover, the excessive fission of neuronal mitochondria can trigger a series of pathological reactions, leading to mitochondrial ultrastructural and morphological damage, oxidative stress, dysfunction, and neuronal degeneration and death [45, 46]. Drp1 is a cytoplasmic protein with a GTPase domain at its N-terminal. As one of the key proteins in mitochondrial dynamics, Drp1 is vital for maintaining mitochondrial morphology and functions [47, 48]. An abnormal increase in Drp1 protein expression beyond the normal range can lead to increased mitochondrial division and membrane permeability, which results in the activation of the endogenous mitochondrial apoptosis pathway and causes apoptosis. SOX2 is an important transcription factor and plays an important role in neuronal development and biogenesis.

Gui et al. [49] reported that SOX2 has a major effect on learning, memory, and PND. Consistent the results of previous studies [40, 49, 50], our findings confirmed that the cognitive decline in mice after surgery is related to the decrease in SOX2 expression. In this study, after knockdown of *SOX2OT* with lentivirus, we found that the expression of SOX2 increased, whereas the expression of Drp1 decreased. In addition, mitochondrial dysfunction, oxidative stress and cognitive function were alleviated to varying degrees. We then used siRNA virus to interfere with the expression of SOX2. The results showed that the expression of Drp1 increased, accompanied by an increase in LPS level, and the mitochondrial membrane potential decreased. Moreover, we found that the knockdown of SOX2 increased LPS-induced mRNA and protein levels of Drp1, indicating that SOX2 regulated Drp1 expression at the transcriptional level. Our luciferase reporter assay showed that SOX2 regulated Drp1 transcription by directly binding to the region of the Drp1 promoter.

This study has several limitations. First, we did not analyze the mitochondrial respiratory chain or oxidative stress markers. Second, only the hippocampus of aged mice was investigated; thus, it is unclear whether these changes would also occur in younger mice or other brain regions. Despite these limitations, we believe that this study provides novel information for understanding the effect of mitochondrial dynamics in PND.

## Conclusions

*SOX2OT* expression is activated in elderly mice with tibial fractures, resulting in mitochondrial and cognitive function deficits. *SOX2OT* knockout reduces the transcription of Drp1 by upregulating the expression of SOX2 and reduces oxidative stress, mitochondrial division, and cognitive dysfunction. The results show that regulating mitochondrial oxidative stress by targeting Sox2/Drp1 is a potential therapeutic strategy for PND.

## Supplementary Information

**Additional file 1:** Drp1 promoter sequence.

**Additional file 2:** Differentially expressed LncRNAs between the surgery and the sham group.

## Data Availability

The datasets used and/or analyzed during the current study are available from the corresponding author on reasonable request. Raw data and processed lncRNA data are included in the Additional files.

## Abbreviations

ANOVA: Analysis of variance
GO: Gene Ontology
KEGG: Kyoto Encyclopedia of Genes and Genomes
NOR: Novel object recognition
OCR: Oxygen consumption rate
PND: Postoperative neurocognitive disorders
ROS: Reactive oxygen species
SOX2: SRY (sex determining region Y)-box 2
SOX2OT: SOX2 overlapping transcript
CCK-8: Cell counting kit-8
LDH: Lactate dehydrogenase
Drp1: Dynamin-related protein 1
BCA: Bicinchoninic acid
