# Supplementary material for: Tibial fracture surgery in elderly mice caused postoperative neurocognitive disorder via SOX2OT lncRNA in the hippocampus
Source: Mol Brain. 2023 Apr 25;16:36. doi: 10.1186/s13041-023-01024-y (PMC10131420; doi:10.1186/s13041-023-01024-y)
Supplement: Supplementary file 1 — Additional file 1: Drp1 promoter sequence. [file 13041_2023_1024_MOESM1_ESM.docx]

drp1 promoter sequence

>5' Flanking sequence chromosome:GRCm39:16:16176824:16178823:-1

ATCAATTGGAGATAGTACCTGCACCTGAGTTAGGAATGGGGGCCTGTGTCTACTTTCAGA

TTATCTTTGTGTGTGTGTGTGTGTGTGTGTGTGTGTGTGTGTGTGTGTGTGTGTGTGTGT

AATAATATTTAGGCCTCATCCCTTCCTGTTTCTTTCCTCTTATGTCTGATTGCTCTGGGC

AATCCTGGCAGTAATGTACTGGTAGACTTTCTTAAGCACTGCCTGTGGGATTATTTTTTC

TCTTACAAAAAGCTACATGATAATATATAAGACTATATGTGTTCTGTTACCAGAGAAAAG

ATAATTTGAGTAGGAACATATACCATACCAATCTATGACATGTTGGCTTTGGTAATATGT

ATGTATATCAATTTTACCAAACTAGCTCTTAGCACACAGTAAAAGACAAAGGAAAATCAA

GTATAAATGCATCATTTTTCCTAGGGTACTTTATTTATCTCTGGCTAGGCTGGAGCTCAC

TATGGAGACAATAGAGCTGACTCTTGCCTCTGCCTTCTCAGTCTGAGGTTACATATATGC

TGCTGCACTTGGTTTTGGATTCTGAAATCTTTGTTTTTAAAAGACAAAAGGGAAGGTCAC

AACATTTGCCAAGATGTCCAAAAATCACTTCTGGATCTTCAGAGTCTTTCATTTATGTGT

TTTTCAAAACTTATTAACCAGCCACCATCTGTGTTCTGAACAGTAAAGCCTGTCTCCTAT

GGCATATGTTGCAGGTGAATGGTAAGTAACCCCTCCAGTTATACAATCCACAACTTTGTG

ACTATGAGTGCTCATGGCTATCATGAATAGATAGCAACAAGTTTTGCTTGGCTTTTCACT

CCTCTCAGGCTGGGGAGTTTGTGGCTCTGAGTTACTGTTTTATTTATGTTTAACAGTCTT

AAAATCCAGTATATTGTGATTATTTTGGTACCCTCATTACCCTCTGATATTCCCCCTTCC

TTTCTAGGACTCCTTCTTCCCACCTATTAGTCCTAGTGGGTATACTAATAGAAAAAGCTA

ATCCCCAACTCCAGCAAACATTCACAGTTACTGCTCCTTTAGAAAGAATGGAGCTTCATG

GGCACCTCCTGTTTCTATGATAGATAGCTGGAGGGCCCAATCTTGTGCAGGTATATAGTG

CTATGTATTCAGGCTACAACTACAGCTTTGTAAGTAGATGACAGCTGCTCAATCCTTGGT

TTCTTATATTCTTTCTGCCTTCATTTCTGCATTGTACCTTAAGTCGTAGTTGGTGTGTGT

GATACGAATGTCTAGTTCTAAGGGCTTATCATTCACTCTCAGAACTTTGACCAGATAAAC

TCTCTTGGTGGGTAGGCTGGAGCGGTAGAAATGTCTGGAGACATTCTTTTAAGTTTTCCT

ATAGTTTCCTTGATTGCTCTTCATAAAGTAGTACTTCTCATAAGGAAATGACATTGGTTA

ATTAATAGACTCTATCCCTATTTTGGAAGGGGGGGGGGGTTCGAGACAGGGTTTCTCTGT

GTAGCCCTGGCTGTCCTGGAACTCATTCTGTAGACCAGGCTAGCCTCGAACTCAGAAATT

CGCCTGCCTCTGCCTCCCAAGTGCTGGGATAAAGGCATGCGCCATCACTGCCCGGCGACT

CTATCCCTTCTTATAAACACAGGATTTCACTATGTAGTACAGACCTTGTTAACCTTTTGT

CTAAGTCTTCCATGGTTGTCCATAAAATTGGTGTGTCCCAACTCCTGGAGGCATACTCAT

ACATAACACTCTAACAATCATTATTCATCTAGCTGTTAGCTAGCATTTTGACTATAGTAG

ACTCAGATTCACGGTCTCAGCTTCCTTACCCGCCCAGTTCAGGTCGATACCTGAAACCTT

CTAGAATAGGCCTTCGCGTTCCACTCAGCCTGAACTACATCTCCCGGCAGCCTTTGCGCG

CCCACCTCTGTCGTGGCGCGTGGCCTGCCGGGAGCGGGCTGGGTGCTGGCCCGGCCCAAT

GGGTGCGGGCTCCCGAGGAA
